# Temperature-Dependent Compensation Points in Gd_x_Fe_1−X_ Ferrimagnets

**DOI:** 10.3390/ma18061193

**Published:** 2025-03-07

**Authors:** Chao Chen, Cuixiu Zheng, Shanshan Hu, Jianwei Zhang, Yaowen Liu

**Affiliations:** School of Physics Science and Engineering, Tongji University, Shanghai 200092, China; 1910784@tongji.edu.cn (C.C.); zhengcuixiu@tongji.edu.cn (C.Z.); hushanshan@tongji.edu.cn (S.H.)

**Keywords:** ferrimagnetism, micromagnetic simulation, spintronics

## Abstract

Recent experiments have reported distinct handedness of spin waves across the compensation temperatures of ferrimagnets, offering promising functionalities for ferrimagnet-based magnonic applications with two distinct polarizations. This paper investigates the effects of various factors on the compensation points of GdFe ferrimagnets through atomistic-level spin dynamics simulations. The results show that as the Gd composition increases, both the magnetization compensation temperature and the angular momentum compensation temperature of the GdFe alloy increase, with a linear relationship observed between the two compensation temperatures. Furthermore, we show that external magnetic fields and antiferromagnetic exchange strength can also modulate the compensation temperatures. Moreover, the antiferromagnetic exchange strength also affects the resonance frequency of ferrimagnetic materials. In the absence of an external field, the resonance frequency of GdFe is divided into two branches and both increase linearly with the increase in antiferromagnetic exchange strength. This study may stimulate fundamental research on compensated ferrimagnets, which may be useful for building chirality-based spintronics.

## 1. Introduction

The spin dynamics of magnetic-ordered systems is a crucial research area. In recent years, with the progress of research technologies and theoretical studies, remarkable achievements have been made in exploring spin dynamics [1,2,3,4,5,6,7,8]. Among various research objects, antiferromagnetic materials have drawn researchers’ attention due to their unique properties. Their high-frequency behavior is special, reaching the terahertz frequency range. Research shows that this high-frequency characteristic is closely related to the antiferromagnetic exchange coupling mechanism [9,10,11,12,13,14,15,16,17,18]. However, antiferromagnetic materials face challenges in practical applications and further research. Their strong resistance to external magnetic fields makes it difficult to accurately control their spin order. Also, during magnon excitation and detection, this resistance causes issues like signal interference and reduced detection accuracy.

In contrast, ferrimagnetic materials offer new approaches to address the issue of controlling antiferromagnetic spin waves. Ferrimagnetic materials possess a net magnetic moment internally, and this distinctive feature endows them with significant advantages in spin-wave manipulation. The net magnetic moment arises from the disparity in the lengths of the magnetic moments within its two sublattices. Despite this difference appearing slight, it exerts a vital influence on the material’s macroscopic magnetism. Through the application of an external magnetic field, the spin dynamics in ferrimagnetic materials can be effectively regulated and precisely adjusted [7,19,20,21]. Among them, rare-earth (RE) and transition-metal (TM) ferrimagnetic alloys such as Gd_x_(FeCo)_1−x_ (0 < x < 1) hold promising applications in magneto-optical recording, heat-assisted magnetic recording (HARM), and all-optical switching (AOS) [22,23,24,25,26,27,28,29,30,31,32]. In these ferrimagnetic amorphous alloys, the transition metal (Fe and Co) forms one sublattice, consisting of both atomic 3d and mobile s-p spins, while the rare-earth metal Gd forms a second sublattice with oppositely aligned spins, primarily localized in the 4f shell. Specifically, the two sublattices provide two particular temperature indicators. One is the magnetization compensation temperature (T_M_). At this temperature, a special change takes place in the internal magnetization state of the material, which is caused by the interaction of magnetization intensities between different sublattices reaching an equilibrium state. The other is the angular-momentum compensation temperature (T_A_). At this temperature, the angular momenta of the sublattices also attain equilibrium. Notably, at T_A_ point, the properties of ferrimagnets are similar to those of antiferromagnetic materials, but the net magnetization is nonzero. The net magnetization and angular momentum exhibit parallel and antiparallel configurations crossing these two compensation points. In contrast to the situation in ferromagnets, where only the right-handed chirality of magnons (the quanta of spin waves) can be supported, ferrimagnets possess both right-handed (RH) and left-handed (LH) magnon modes. This characteristic endows an additional fascinating dimension for the manipulation of spin order, that is, the chirality or handedness of spin waves. Besides the amplitude and phase of spin waves, this chirality can also be exploited for information transmission. Depending on magnetization precessional directions with the external magnetic field, both the right-handed and left-handed spin wave chiralities are permitted in ferrimagnets. Recent experiments have reported the RH and LH excitation of spin waves across the two compensation temperatures of ferrimagnets, such as GdCo [33], Gd_3_Fe_5_O_12_ [34], and artificial ferrimagnetic multilayers [35]. Recently, our previous work show that the chirality switching could also be observed by changing the composition of ferrimagnets to cross the compensation points of magnetization and angular momentum [36]. All these studies facilitate the realization of antiferromagnetic magnonic devices equipped with the dual handedness of spin waves that can operate at ultrahigh speed [37]. The ability to modulate the magnetic moment compensation point of ferrimagnetic materials through external conditions opens up significant potential for the development of new spintronic devices with tailored magnetic properties.

This article mainly investigates the changes in magnetic compensation points and angular momentum compensation points in the ferrimagnetic alloy Gd_x_Fe_1−x_, with respect to factors such as the Gd composition ratio (*x*), external magnetic field, and antiferromagnetic exchange strength between the RE and TM atoms. Our results indicate that, as the Gd ratio increases, both the magnetic compensation temperature and the angular momentum compensation temperature increase significantly, with a linear relationship observed between the two compensation temperatures. In addition, the magnetic compensation temperature increases with the higher external magnetic field or stronger antiferromagnetic exchange coupling variation. These findings provide promising potential for controlling the magnetic compensation points in ferrimagnetic materials. Furthermore, we delved into the influence of the antiferromagnetic exchange intensity on the resonance frequencies of the GdFe alloy and closely monitored the alterations in its chirality. Through in-depth research, it was revealed that GdFe alloys exhibit two distinct resonance frequencies: a high-frequency mode and a low-frequency mode. Intriguingly, the chirality associated with the high-frequency mode is left-handed, while that of the low-frequency mode is right-handed. This discovery has made a substantial contribution to our comprehension of the chirality characteristics in ferrimagnetic materials, thereby deepening our knowledge in this area of magnetic material research.

## 2. Atomistic-Level Simulation Model

**Figure 1:**
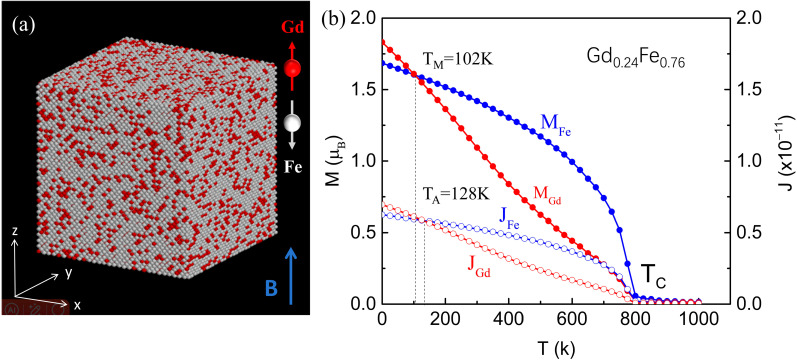
(**a**) Illustration of atomistic simulation model for the Gd_x_Fe_1−x_ ferrimagnetic alloy. The magnetic moments of Gd and Fe atoms are antiparallel in the ground state. *B* is the external magnetic field applied in *z*-axis direction. (**b**) The calculated magnetic moments of sublattice Gd (red solid circles with line) and Fe sublattice (blue solid circles with line) as functions of temperature for a given concentration of *x* = 0.24. The open colored circles with lines show the corresponding angular momenta of the two sublattices. The two crossing points between the curves indicate the magnetization compensation temperature (T_M_) and angular momentum compensation temperature (T_A_), respectively.

In this study, we focus on the ferrimagnetic alloy Gd_x_Fe_1−x_ within the RE-TM system, and its structure is shown in Figure 1a. The unique magnetic properties of the ferrimagnetic alloy possess great application potential in the fields of spintronics and the development of new magnetic materials, thus attracting much attention from researchers. We employ the atomic-scale micromagnetic simulation technique to conduct an in-depth study of the GdFe alloy. During the simulation, the Hamiltonian energy includes the contributions from the Heisenberg exchange, magnetic anisotropy, and the Zeeman effect. The formulas are as follows:

(1) $$H=-\sum_{j<k}J_{jk}m_{j}\cdot m_{k}-K_{u}\sum_{j}m_{jz}^{2}-\mu_{S}\sum_{j}B_{ext}\cdot m_{j}$$

where **m***_j_* is the unit vector describing the local spin moment direction at the *j*th lattice site, and the atomic spin moment $\mu_{s}$ is related to the macroscopic saturation magnetization $M_{s}$ of the sample by $\mu_{s}=M_{s}a^{3}/n_{\mathrm{at}}$. Here, a is the unit cell size, and $n_{\mathrm{at}}$ is the number of atoms per unit cell. *J_jk_* denotes the exchange interaction between two neighboring magnetic atom sites. The Gd_x_Fe_1−x_ exhibits perpendicular magnetic anisotropy (PMA), characterized by a PMA constant *K_u_* with the *z*-axis as its easy axis. **B**_ext_ represents the external magnetic field. The dynamics of the macroscopic magnetization **m** are routinely described by the Landau–Lifshitz–Gilbert (LLG) equation [38]. In a ferrimagnetic system, the LLG equation can be written for the *i*th sublattice (*i* = RE, TM) as follows [39,40,41]:

(2) $$\frac{dm_{i}}{dt}=-\left|\gamma_{i}\right|(m_{i}\times B_{\mathrm{eff}}^{i})+\frac{\alpha_{i}}{m_{i}}(m_{i}\times \frac{dm_{i}}{dt})$$

where $\gamma_{i}$ is the gyromagnetic ratio for the *i*th sublattice, and $B_{\mathrm{eff}}^{i}$ is the effective field acting on the ith sublattice. The gyromagnetic ratio $\gamma_{i}$ and the Gilbert damping parameter $\alpha_{i}$ are given by

(3) $$\left|\gamma_{i}\right|=g_{i}\frac{\mu_{B}}{\hbar },\;\;\;\;\alpha_{i}=\frac{\lambda_{i}}{\left|\gamma_{i}\right|m_{i}}$$

where $\mu_{B}$ is the Bohr magnon, $g_{i}$ is the Landé g factor, and ℏ is the reduced Planck constant. λ is the Landau–Lifshitz damping parameter [39]. These equations are coupled by the presence of the exchange field $H_{RE,\;TM}^{ex}=-\lambda_{ex}m_{RE,TM}$ between the RE and TM sublattices. The resonance frequencies are obtained with the following: $\omega_{\pm }=\gamma \mu_{0}(H_{SF}\pm H_{0})$, where $H_{SF}=\sqrt{2H_{RE,\;TM}^{ex}H_{RE,\;TM}^{k}+{H_{RE,\;TM}^{k}}^{2}}$ is the spin-flop (SF) field, and $H_{RE,\;TM}^{k}$ is the magnetic anisotropy field. The effective gyromagnetic ratio $\gamma_{eff}$ for a ferrimagnet can be described as follows [22]:

(4) $$\gamma_{eff}(T)=\frac{m_{Gd}(T)-m_{Fe}(T)}{J_{Gd}(T)-J_{Fe}(T)}=\frac{M(T)}{A(T)}$$

where $J_{Gd}=m_{Gd}/|\gamma_{Gd}|$ and $J_{Fe}=m_{Fe}/|\gamma_{Fe}|$ represent the angular momenta of Gd and Fe sublattices. *M*(T) and *A*(T) are the net magnetic moment and net angular momentum, respectively. Because the two magnetic sublattices of a ferrimagnet sample are arranged antiparallel to each other, but their magnetic moment magnitudes are unequal, resulting in a net magnetization at low temperatures. As the temperature increases, the magnetization of the two sub lattices changes at different rates, giving rise to two characteristic points: the magnetization compensation temperature (T_M_) and the angular momentum compensation temperature (T_A_). According to Equation (4), T_M_ is defined as the temperature at which the magnetizations of the two sublattices are equal in magnitude but opposite in direction, i.e., $\left|\sum m_{Gd}\right|=\left|\sum m_{Fe}\right|$. Meanwhile, T_A_ corresponds to the temperature at which the angular momenta of the two sublattices are equal, with the gyromagnetic ratio γ depending primarily on the Landé *g*-factors.

In our simulations, the relevant material parameters for GdFe are taken as follows [42]: the Gilbert-damping constant is *α* = 0.02, the neighboring exchange fields between atoms are $J_{Fe-Fe}=4.5\times 10^{-21}\;J/link$, ${\;J}_{Gd-Gd}=1.26\times 10^{-21}\;J/link$, and $J_{Fe-Gd}=-1.09\times 10^{-21}\;J/link$, respectively. The atomic spin moments are $\mu_{Fe}=2.217\mu_{B}$ and $\mu_{Gd}=7.63\mu_{B}$. The PMA constant is ${k_{u}=8.07246\times 10}^{-24}\;J/link$. The Landé g factors for Fe and Gd are $g_{Fe}=2.0$ and $g_{Gd}=2.05$, respectively.

## 3. Results

In this study, we first use atomistic-level micromagnetic simulations to investigate the T_M_ and T_A_ of the Gd_0.24_Fe_0.76_ sample. The model comprises randomly distributed Fe and Gd atoms, as illustrated in Figure 1a. Through simulations, we obtain the temperature-dependent behaviors of magnetic moments (solid colored squares with lines) and angular momenta (open colored squares with lines) for the Fe and Gd sublattices, as shown in Figure 1b. The crossing points of the magnetic moment curves indicate the magnetic compensation temperature T_M_ = 102 K, where $\left|\sum m_{Gd}\right|=\left|\sum m_{Fe}\right|$. Similarly, the crossing point between *J*_Gd_ and *J*_Fe_ curves shows the angular momentum compensation point T_A_ = 128 K, where $\left|\sum J_{Gd}\right|=\left|\sum J_{Fe}\right|$. Between the T_M_ and T_A_ points, the angular momentum vector is parallel to magnetic moment, with a negative gyromagnetic ratio γ of electronic spin.

**Figure 2:**
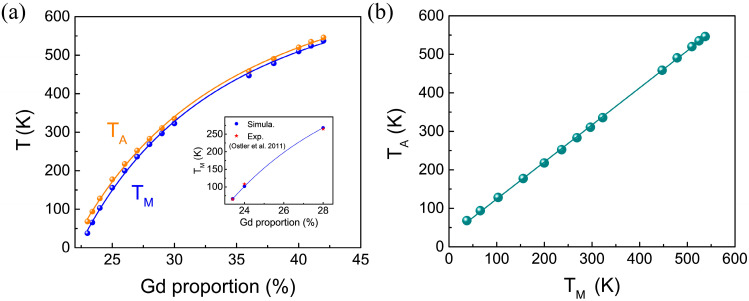
(**a**) The calculated magnetization compensation temperature T_M_ and angular momentum compensation temperature T_A_ as functions of different Gd proportions. The inset plot shows the comparison of the T_M_ between our simulation and experimental data taken from Ref. [6]. (**b**) Linear relationship between T_M_ and T_A_, fitted by a linear function of T_A_ = 0.85TM + C.

We further simulated the variations in the magnetic compensation point T_M_ and the angular momentum compensation point T_A_ in Gd_x_Fe_1−x_ alloys as a function of the Gd component. The results are shown in Figure 2a. Notably, the calculated T_M_ values at *x* = 23.4%, 24%, and 28% are in excellent agreement with experimentally measured magnetic compensation temperatures [6], further validating the reliability of our simulation model. As shown in Figure 2a, the minimal variation in the Landé g factors of Fe and Gd atoms results in a very small difference between T_M_ and T_A_. Interestingly, while T_M_ and T_A_ increase with higher Gd concentrations, their relationship is not strictly linear. However, a surprising observation is that, despite the nonlinear dependence on Gd concentration, T_M_ and T_A_ exhibit a clear linear relationship with each other when analyzed as functions of temperature, as shown in Figure 2b.

**Figure 3:**
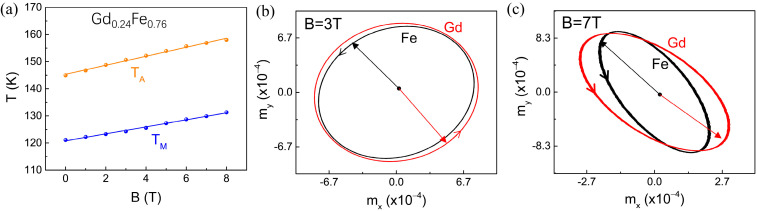
(**a**) The calculated magnetization compensation temperature (T_M_) and angular momentum compensation temperature (T_A_) as functions of external magnetic field for a fixed concentration of Gd_0.24_Fe_0.76_. (**b**,**c**) The observed precession trajectories of the resonance mode at external magnetic fields of 3T and 7T, respectively. The trajectories of Fe and Gd sublattices are projected onto the xy-phase plane. Where red represents the trajectory of Gd sublattices and black represents the trajectory of Fe sublattices.

The external magnetic field could significantly change the magnetic compensation temperature T_A_ and the angular momentum compensation temperature T_M_. The simulated T_M_ and the T_A_ as functions of magnetic field for a fixed concentration of Gd_0.24_Fe_0.76_ are shown in Figure 3a, and the result reveals that both the T_M_ and the T_A_ exhibit a nearly linear increase with increasing magnetic field strength. (Approaching the proportion for angular momentum compensation serves as a promising platform for exploring and manipulating the antiferromagnetic spin order.) This result indicates the significant influence of the magnetic field on the dynamics of the ferrimagnetic system, making it a viable control parameter for tuning the compensation points. To further elucidate this behavior, the precession trajectories of Fe and Gd sublattices are plotted at the resonance frequency of 260 GHz (under a 3 T magnetic field) and 350 GHz (under a 7 T magnetic field), as shown in Figure 3b and Figure 3c, respectively. These trajectories, projected in the xy-plane, highlight the dynamic behavior of sublattice moments under two different magnetic field strengths. The external magnetic field influences the orientation of the magnetic moments in GdFe ferrimagnetical alloys. Before reaching the magnetic compensation temperature, the external magnetic field induces the magnetic moments to deflect towards the direction of the magnetic field. As the magnetic field strength increases, the degree of this deflection becomes more pronounced, making it increasingly difficult for the overall magnetic moments to achieve a fully compensated (canceled) state. Consequently, the magnetic compensation temperature rises because a higher temperature is required to rebalance and compensate for the magnetic moments.

A strong correlation exists between the magnetic compensation temperature and the angular momentum compensation temperature. When the external magnetic field changes one of these two compensation temperatures, the other is also indirectly affected. This interdependence arises because both T_M_ and T_A_ are fundamentally determined by the magnetic structure and the electron spin states of ferrimagnets. The magnetic field influences the magnetic moments and angular momentum through internal interactions, leading to synergistic changes in both compensation temperatures. However, it is important to note that the efficiency of controlling compensation temperatures via external magnetic fields is significantly lower than that achieved by varying the proportion of the Gd component. This highlights the critical role of composition tuning in precisely regulating the compensation temperatures of ferrimagnetic materials.

**Figure 4:**
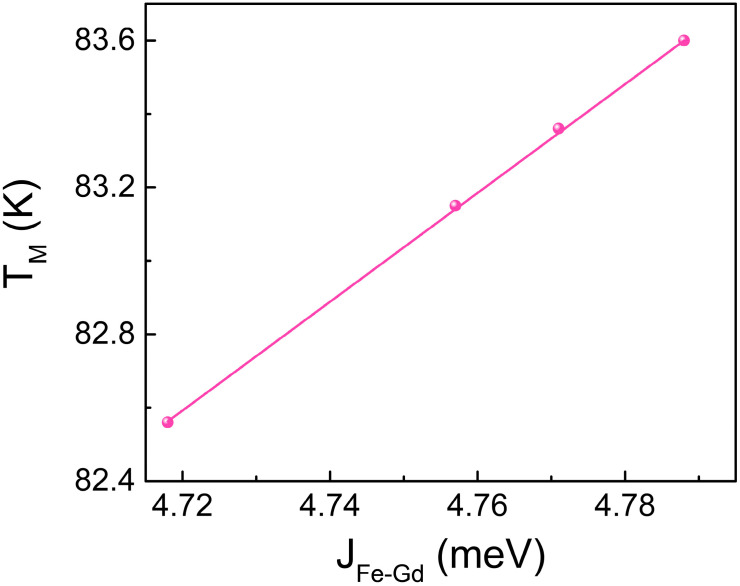
The calculated magnetization compensation temperature (T_M_) as a function of sublattice exchange coupling strength J_Fe-Gd_ between the Fe and Gd atoms.

We also investigated the influence of inter-sublattice exchange coupling on the magnetic compensation temperature T_M_ in the GdFe ferrimagnetic alloy. The results indicate that T_M_ increases linearly with the enhancement of antiferromagnetic exchange coupling between RE and TM atoms, as shown in Figure 4. A stronger antiferromagnetic exchange coupling enhances the alignment between magnetic moments, changes their equilibrium state, and consequently raises the magnetic compensation temperature. This occurs because a higher temperature is needed to provide sufficient thermal energy to overcome the enhance antiferromagnetic exchange coupling and achieve the compensation state of the magnetic moments. Furthermore, enhanced antiferromagnetic interaction affects the orientation distribution of magnetic moments, resulting in a more ordered magnetic alignment. At the magnetic compensation temperature, additional thermal energy is required to disrupt this orderly arrangement and achieve a state where the magnetic moments cancel each other, and thus an increase in T_M_.

**Figure 5:**
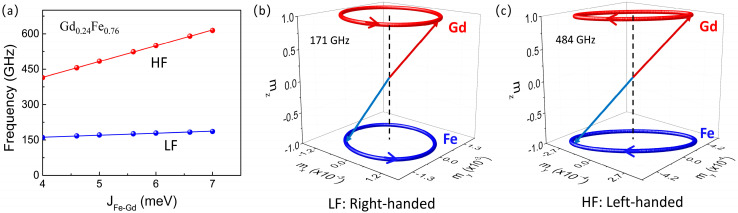
(**a**) The resonance frequency as a function of sublattice exchange coupling strength J_Fe-Gd_ between the Fe and Gd atoms. (**b**) The magnetization precession trajectory of low-frequency mode when J_Fe-Gd_ = 5 meV, with right-handed chirality (**c**) The magnetization precession trajectory of high-frequency mode when J_Fe-Gd_ = 5 meV, with left-handed chirality.

We further investigated the influence of the exchange coupling J_Fe-Gd_ between Fe atoms and Gd atoms on the resonance frequency. Figure 5a shows the resonance frequency as a function of J_Fe-Gd_, where a microwave excitation field $h_{\mathrm{mw}}\left(t\right)=h_{0}\sin(2\pi f_{\mathrm{mw}}t)$ with an amplitude of $h_{0}=\;5\;\mathrm{mT}$ and a driving frequency of $f_{\mathrm{mw}}$ is applied. Obviously, the observed resonance modes can be divided into a high-frequency mode (HF) and a low-frequency mode (LF). For a typical collinear antiferromagnetic sample, when no external magnetic field is applied, the resonance modes should be degenerated. However, for the ferrimagnetic alloy Gd_0.24_Fe_0.76_, at this composition ratio, the magnetization of Fe atoms and Gd atoms cannot be completely compensated. Therefore, there exist two modes: high frequency and low frequency. In addition, it can be observed that the resonance frequencies of both modes increase linearly with the increase in J_Fe-Gd_. The increase rate of the high-frequency mode is much greater than that of the low-frequency mode. Moreover, the HF exhibits left-handed chirality, while the LF exhibits right-handed chirality, as shown in Figure 5b,c. For typical collinear antiferromagnetic samples, the resonance frequency is also divided into high-frequency mode and low-frequency mode during the application of an external magnetic field. However, the high-frequency mode exhibits right-handed chirality, while the low-frequency mode exhibits left-handed chirality. In the ferromagnetic alloy GdFe, the chirality of high-frequency and low-frequency modes will switch due to the different external magnetic fields. We predict that, in our material, if a large external magnetic field is applied, the chirality of HF and LF will reverse [36].

## 4. Conclusions

In summary, we investigated the factors influencing the magnetic compensation temperature T_M_ and the angular momentum compensation temperature T_A_ in the ferrimagnetic alloy GdFe. Our simulations demonstrate that the Gd composition, external magnetic field, and inter-sublattice exchange strength can all play significant roles in tuning T_M_ and T_A_. The magnetization compensation temperature and the angular momentum compensation temperature both increase proportionally with Gd composition, and a linear relationship exists between T_M_ and T_A_. Additionally, the external magnetic field, inter-sublattice exchange strength, and strain exhibit a proportionality with the magnetic compensation temperature. The compensation temperatures of T_M_ and T_A_ are critical parameters that characterize the unique magnetic properties of ferrimagnetic materials. These temperatures not only reveal the intricate microscopic magnetic structures and electron spin interactions within the materials but also offer immense potential for technological applications in fields such as magnetic storage, magneto-optical devices, and antiferromagnetic spintronics. In addition, we also studied the effect of interlayer coupling between Fe atoms and Gd atoms on the resonance frequency and their chirality changes. The simulation results showed that, in the absence of an external magnetic field, the resonance frequency of Gd_0.24_Fe_0.76_ alloy was divided into two categories: high-frequency mode and low-frequency mode, with the high-frequency mode exhibiting left-handed chirality and the low-frequency mode exhibiting right-handed chirality. This discovery not only broadens our understanding of the chirality in ferrimagnetic materials but also serves as a solid foundation for the design of chirality-based devices.

## Data Availability

The original contributions presented in this study are included in the article. Further inquiries can be directed to the corresponding authors.
